# Compressed Branching Geometry in a Bovine-Type Aortic Arch: A Morphometric Cadaveric Case Report and Systematic Review of the Literature

**DOI:** 10.7759/cureus.117241

**Published:** 2026-09-30

**Authors:** Adrian A Naoun, Hector L Figueroa Monsanto, Wilson Veras

**Affiliations:** 1 Medicine, San Juan Bautista School of Medicine, Caguas, PRI

**Keywords:** aortic arch variant, bovine aortic arch, brachiocephalic trunk, cadaveric case report, morphometric variation

## Abstract

Anatomical variation in aortic arch branching is common and clinically significant, yet specimen-level morphometric characterization of a bovine-type aortic arch (BAA) remains scarce. We report a two-branch bovine-type arch that was identified during cadaveric dissection of a formalin-embalmed male decedent in his late 70s. The left common carotid artery arose proximally from the brachiocephalic trunk, which then bifurcated into the right common carotid and right subclavian arteries; the left subclavian artery arose separately from the arch. We contextualized this finding by conducting a systematic review of cadaveric studies reporting the same configuration. Five database sources yielded 191 records, of which 14 cadaveric studies met the eligibility criteria and were appraised using two Joanna Briggs Institute (JBI) critical-appraisal instruments.

The specimen's brachiocephalic trunk measured 18.73 mm externally, which falls within the 18.39-18.84 mm external-diameter range reported in the bovine cadaveric literature. Its luminal diameter measured 17.1 mm, which was also within the corresponding 16.85-17.35 mm range. This caliber may reflect a vessel accommodating combined bilateral carotid perfusion, rather than a pathological dilatation. The single interostial distance between the brachiocephalic trunk and the left subclavian artery measured 7.18 mm externally and 8.5 mm endoluminally. The endoluminal measurement was 34.2% below the 12.91 mm reported in a prior cadaveric BAA specimen and 11.5% below the 9.6 mm mean reported in the only bovine cadaveric series that reported this parameter, with both measured endoluminally. The measured carotid take-off length of 8.01 mm fell within, but near the lower limit of, the range reported across five previously published cadaveric bovine studies (6.0-17.3 mm).

The mid-arch diameter measured 25.18 mm, 16.1% below the reported male mean that was obtained at the corresponding anatomical plane using outer-wall measurements. The arch showed proximal-to-distal tapering without aneurysmal ectasia, mural thrombus, or atherosclerotic calcification. Four of the prior included studies reported BAA-specific morphometry that could be interpreted against a stated convention. The reported interbranch distances varied by more than sixfold; heterogeneous measurement methodologies likely contributed substantially to this apparent variability, as most studies did not define their anatomical landmarks. Harmonizing three series with disparate measurement conventions to a common landmark framework yielded closely concordant interbranch distances. The compact BAA branching geometry observed here may be relevant to the available anatomical corridor during arch and carotid interventions, although clinical consequences cannot be inferred from this limited dataset. Standardized measurement protocols with explicitly defined anatomical landmarks are therefore necessary to enable meaningful cross-study morphometric comparisons.

## Introduction

The aortic arch displays a heterogeneous branching pattern that carries substantial clinical implications [1]. In the most common human configuration, three supra-aortic vessels arise independently from the arch: the brachiocephalic trunk, the left common carotid artery, and the left subclavian artery [2]. The brachiocephalic trunk subsequently bifurcates into the right common carotid and right subclavian arteries, while the left common carotid and left subclavian arteries arise separately from the arch [2]. The variant traditionally known as the bovine-type aortic arch (BAA) includes either a common brachiocephalic-left common carotid origin or a proximal left common carotid origin arising from the brachiocephalic trunk [1,2].

Embryologically, the aortic arch develops from the aortic sac, the left fourth pharyngeal arch artery, and the adjacent segment of the left dorsal aorta [3]. Variant branching patterns, including the BAA, are thought to arise from altered resorption or asymmetric remodeling during the establishment of the principal aortic branches in embryonic weeks 5-6 [3]. In a meta-analysis of 23,882 adult arches, BAA configurations accounted for approximately 13.6% of individuals overall, with prevalence reaching 26.8% in some populations [3]. A separate systematic review of 4,609 cadaveric arches reported a comparable two-branch prevalence of 13% [1]. Although widely cited in the anatomical and clinical literature, the term "bovine" is misleading, as this configuration does not occur in cattle [1,2].

The clinical relevance of BAA variants stems from their potential to alter supra-aortic hemodynamics, vessel-wall stress, and susceptibility to aneurysmal remodeling [4,5]. A common carotid origin combined with a compact, relatively horizontal arch configuration may inherently increase technical complexity during open, endovascular, and transcatheter interventions [6,7]. Notably, pathology involving a brachiocephalic trunk that supplies both carotid circulations as well as the right vertebral circulation may lead to extensive cerebral ischemia [8]. It is conceivable that its broader perfusion territory accounts for a larger caliber compared with a conventional right-sided brachiocephalic trunk [8]. Reported prevalence varies substantially across studies, in part because a true common trunk is not consistently differentiated from closely approximated V-shaped ostia that may appear confluent [9,10,11].

Although the prevalence of BAA is well documented in imaging and cadaveric series, standardized specimen-level morphometric data remain limited. To provide context for the present specimen, we conducted a systematic review of cadaveric studies reporting BAA morphometry (the search strategy appears in the Discussion and Appendix). A systematic search of five databases identified fourteen cadaveric studies that documented a bovine-type configuration involving the brachiocephalic trunk and left common carotid artery. Only a minority reported measurements specific to the BAA configuration, and two prior cadaveric reports offered potentially comparable interostial measurements [8,12].

In this report, we describe the morphometry of a two-branch BAA identified in a formalin-embalmed male cadaver in his late seventies and place these findings within a systematic review of specimen-level BAA morphometry. Our objectives were as follows: to characterize the specimen's branching geometry across multiple parameters; to identify and critically appraise cadaveric studies reporting a bovine-type configuration involving the brachiocephalic trunk and left common carotid artery; and to assess the comparability of reported measurements across studies in accordance with the PRISMA (Preferred Reporting Items for Systematic Reviews and Meta-Analyses) 2020 guidelines [13].

## Case presentation

During routine anatomical dissection, a formalin-embalmed adult male cadaver in his late 70s underwent a thoracic vascular anatomy examination. The specimen’s cause of death, antemortem imaging, history of thoracic trauma, and history of major vascular disease were unavailable. An anterior thoracic dissection approach exposed the mediastinum, aortic arch, and supra-aortic branches. The arch demonstrated a two-branch BAA configuration, with only the brachiocephalic trunk and left subclavian artery arising directly from the aortic arch. The left common carotid artery originated proximally from the brachiocephalic trunk rather than independently from the arch. Accordingly, the brachiocephalic trunk gave rise to the left common carotid, right common carotid, and right subclavian arteries (Figure 1). No separate left common carotid ostium was identified on the aortic arch.

**Figure 1:**
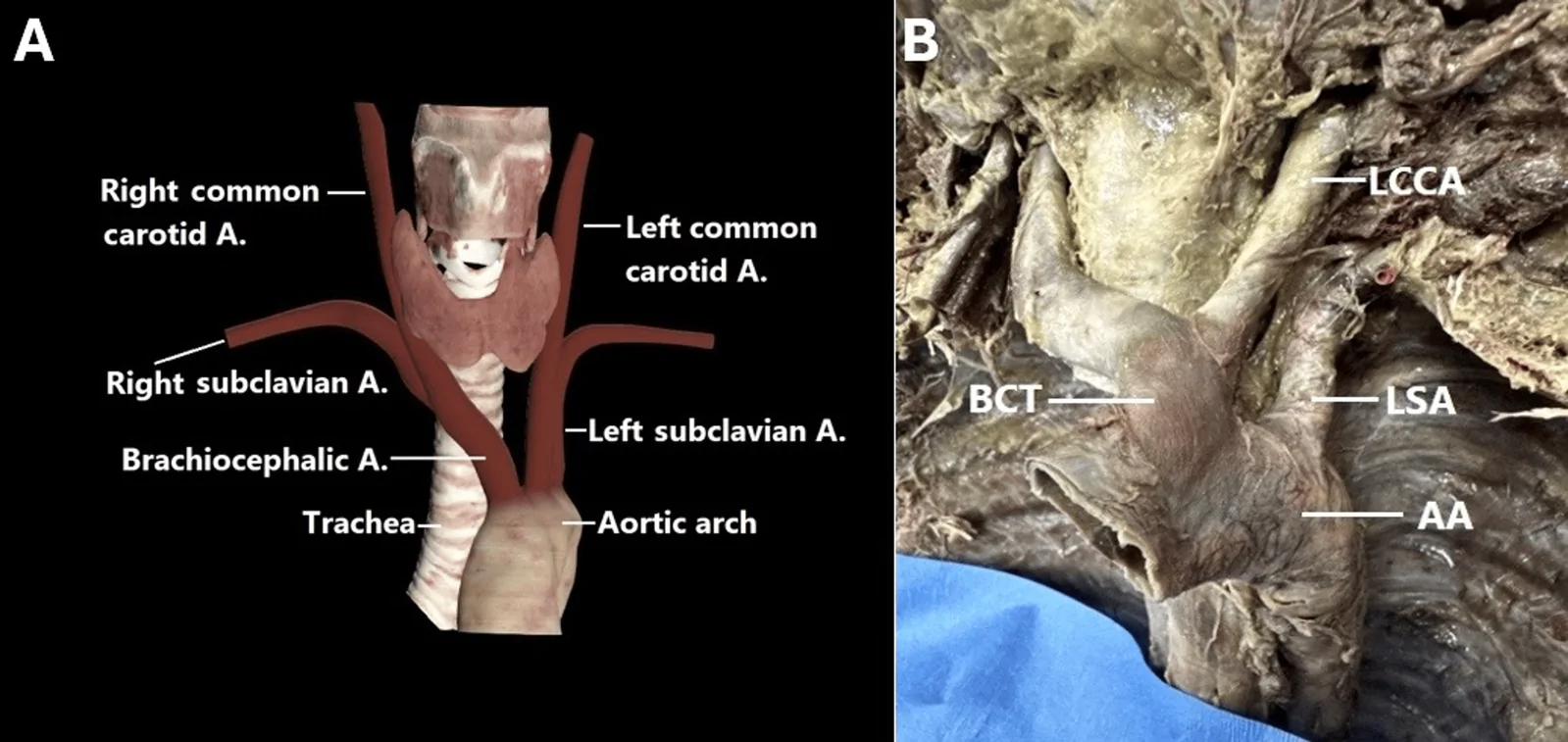
Comparison of expected and observed aortic arch branching (A) Standard three-branch configuration, with separate origins of the brachiocephalic trunk (BCT), left common carotid artery (LCCA), and left subclavian artery (LSA). (B) Anterior mediastinal view of the cadaveric specimen showing the bovine-type two-branch configuration, with the brachiocephalic trunk arising from the anterior aortic arch (AA), passing anterior to the trachea and deviating slightly leftward. Panel A was generated with the Anatomage® Table (Anatomage Inc., Santa Clara, CA), while panel B is a cadaveric specimen photograph taken by the authors

Further evaluation demonstrated that the brachiocephalic trunk originated from the anterior aortic arch and traversed the superior mediastinum, coursing anterior to the trachea with slight leftward deviation. The trunk branched into the left common carotid artery proximally before bifurcating into the right common carotid and right subclavian arteries. The carotid take-off length, defined as the distance from the brachiocephalic ostium to the left common carotid origin, was 8.01 mm. This measurement reflects intratruncal branching length rather than an interostial distance. The trunk followed a near-horizontal trajectory, contrasting with the customary oblique rightward course of a conventional three-branch arch. All measurements were obtained using a digital caliper with a stated resolution of 0.01 mm and were independently performed by two observers (Figure 2).

**Figure 2:**
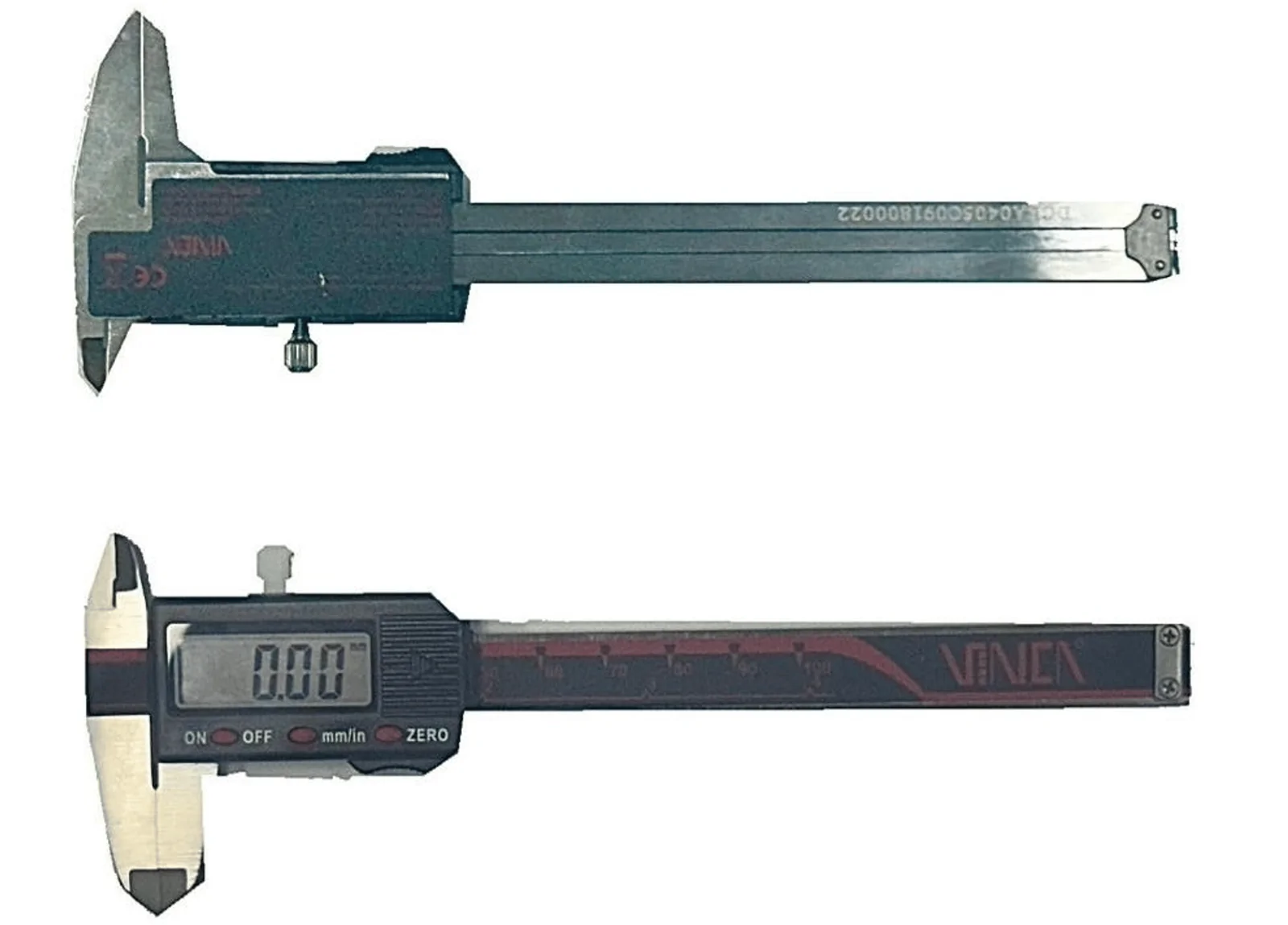
Digital caliper used for all morphometric measurements Complete instrument (Vinca digital vernier caliper; resolution 0.01 mm). All values reported in Table 1 were obtained with this instrument, under the measurement conventions defined in the Case Presentation. Original photograph taken by the authors

Diameters and interostial distance were measured externally on the intact vessel wall and subsequently endoluminally following anterior longitudinal arteriotomy of the aortic arch. Interostial distance was defined as the edge-to-edge interval between adjacent ostial rims. The basis for the difference between the external and endoluminal values is addressed in the Discussion. Morphometric measurements are summarized in Table 1.

**Table 1:** Morphometric measurements of the aortic arch and its branches All measurements were obtained by direct digital caliper assessment of the formalin-embalmed specimen. External values were recorded on the intact vessel wall; luminal and endoluminal values were recorded following anterior longitudinal arteriotomy of the aortic arch. Arch diameters are external. The carotid take-off length reflects intratruncal branching length rather than an interostial distance

Parameter	Measurement (mm)
Proximal aortic arch diameter	28.68
Mid-arch diameter	25.18
Distal aortic arch diameter	24.15
Brachiocephalic trunk diameter, external	18.73
Brachiocephalic trunk diameter, luminal	17.1
Brachiocephalic trunk length, base to right subclavian origin	39.97
Vertical height, arch to right common carotid origin	8.15
Vertical height, arch to left common carotid origin	9.57
Carotid take-off length, brachiocephalic ostium to left common carotid origin	8.01
Interostial interval, trunk to left subclavian, external surface	7.18
Interostial interval, trunk to left subclavian, endoluminal	8.5

The proximal, mid-, and distal aortic arch diameters were 28.68 mm, 25.18 mm, and 24.15 mm, respectively. The mid-arch was defined as the segment between the brachiocephalic trunk and left subclavian artery origins. The brachiocephalic trunk measured 18.73 mm externally and 17.1 mm luminally. The length from the brachiocephalic ostium to the right subclavian origin was 39.97 mm. Vertical distances from the arch to the right and left common carotid origins measured 8.15 mm and 9.57 mm, respectively. The sole interostial interval, between the brachiocephalic trunk and left subclavian artery, measured 7.18 mm externally. Endoluminal measurement through an anterior arch window yielded an interval of 8.5 mm, using the technique described by Babu and Sharma [8]. The arch curvature angle was approximately 60°. The trachea remained midline posterior to the brachiocephalic trunk, consistent with normal mediastinal relationships. No aneurysmal dilatations, mural thrombi, or atherosclerotic calcifications were identified (Figure 3).

**Figure 3:**
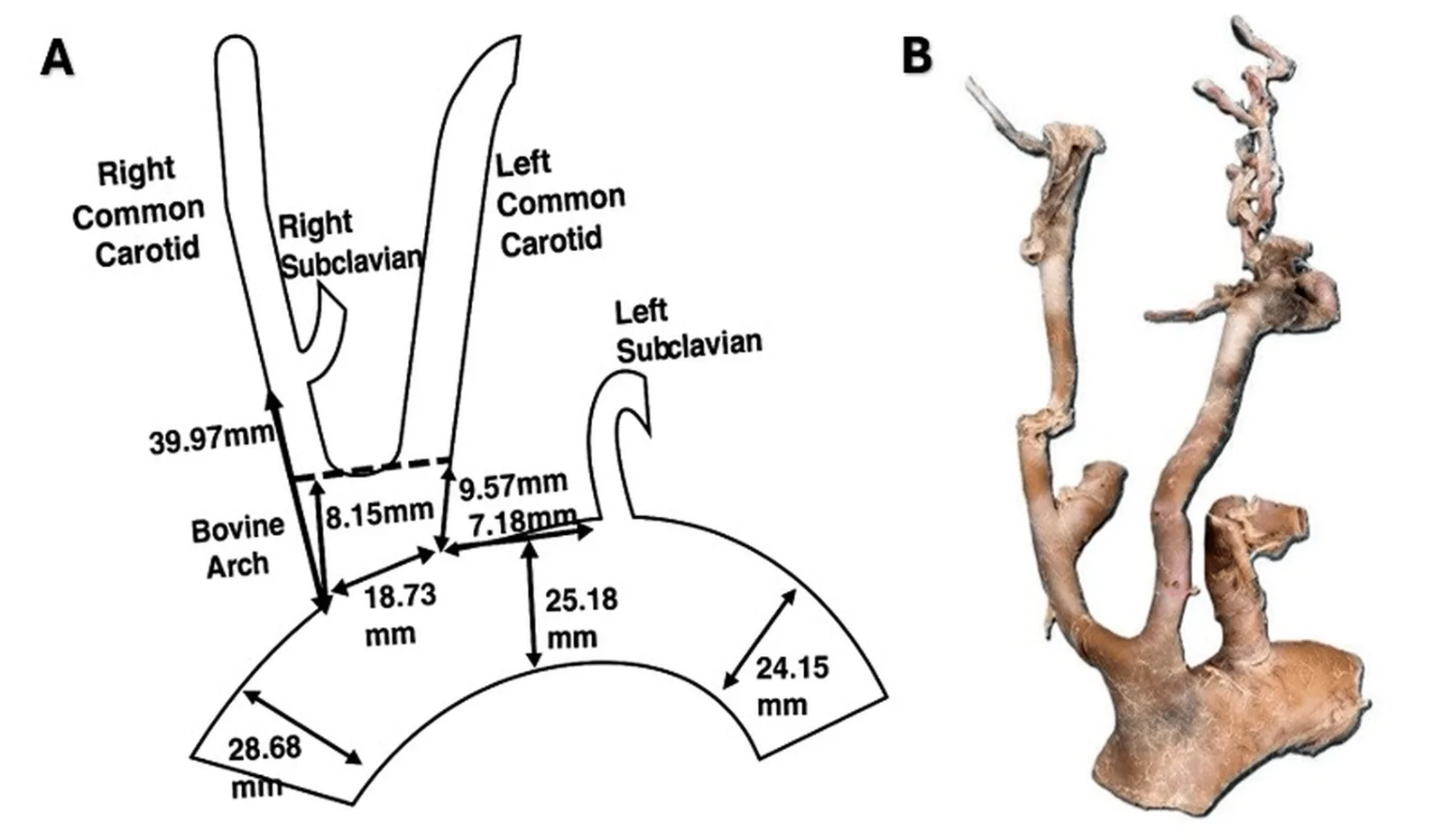
Aortic arch variant with bovine-type branching configuration (A) Schematic representation with morphometric measurements (mm) showing the common origin of the brachiocephalic trunk and left common carotid artery. (B) Isolated aortic arch specimen demonstrating proximal origin of the left common carotid artery from the brachiocephalic trunk and distal bifurcation into the right common carotid and right subclavian arteries; no aneurysmal dilatation, mural thrombus, or atherosclerotic calcification was present. Panel A is an original schematic created by the authors in Microsoft PowerPoint (Microsoft Corp., Redmond, WA), while panel B is a cadaveric specimen photograph taken by the authors

## Discussion

This cadaveric case provides direct morphometric characterization of a bovine-type aortic arch with markedly compressed proximal branching geometry. Comparison with published data was limited by heterogeneity in specimen preparation, anatomical landmarks, and measurement conventions. Notably, most studies did not define their measurement methods explicitly. Accordingly, we conducted a structured systematic review of cadaveric studies documenting a bovine-type configuration involving the brachiocephalic trunk and left common carotid artery. Our objectives were to situate the present specimen within the available evidence and determine whether published morphometric measurements permit direct comparison.

Search strategy and eligibility criteria

We searched five databases from inception to August 2026: PubMed, EBSCOhost, OpenAlex, Google Scholar, and Ovid EBM Reviews. The strategy combined controlled vocabulary and free-text variants for the aortic arch, branching pattern, BAA, cadaveric dissection, and morphometry (Appendix). No language or date restrictions were applied. Non-English reports underwent full-text translation and were retained when eligibility could be confirmed. Records from all databases were pooled and deduplicated before screening.

Eligible studies were primary cadaveric investigations specifically reporting a documented bovine-type configuration involving the brachiocephalic trunk and left common carotid artery, with morphometric measurements of at least one specimen. We excluded imaging-derived studies, non-human specimens, device-based or procedural investigations, conference abstracts, and non-primary reviews. Studies describing anatomically distinct variants, including a bicarotid trunk, were excluded because of their different embryological substrate and implications for endovascular planning. This criterion was applied uniformly during title and abstract screening and full-text review.

Google Scholar was searched separately using three keyword combinations targeting BAA morphometry, cadaveric dissection, and interbranch distance; complete strings are provided in the Appendix section. Searches were performed in a private-browsing session to minimize personalization-related ranking effects. Given Google Scholar’s relevance-ranked output rather than nested Boolean syntax, screening was prospectively limited to the first 100 results for each query. The resulting arm yielded 16 records, which were pooled and deduplicated with records from the other four databases altogether.

Search results and study selection

The search yielded 191 records: 49 from PubMed, 61 from EBSCOhost, 65 from OpenAlex, 16 from Google Scholar, and none from Ovid EBM Reviews. After removal of 56 duplicates, 135 records were screened by title and abstract; 110 were excluded. We sought and retrieved the full texts of all 25 remaining reports. Eleven were excluded at full-text review: two were imaging-derived rather than cadaveric, two additional were systematic classification reviews rather than a primary cadaveric study, and seven did not document the prespecified common brachiocephalic-left common carotid origin. The latter group included two reports of a bicarotid trunk only, defined by a common origin of the common carotid arteries. Fourteen studies met all eligibility criteria and were included in the synthesis (Figure 4).

**Figure 4:**
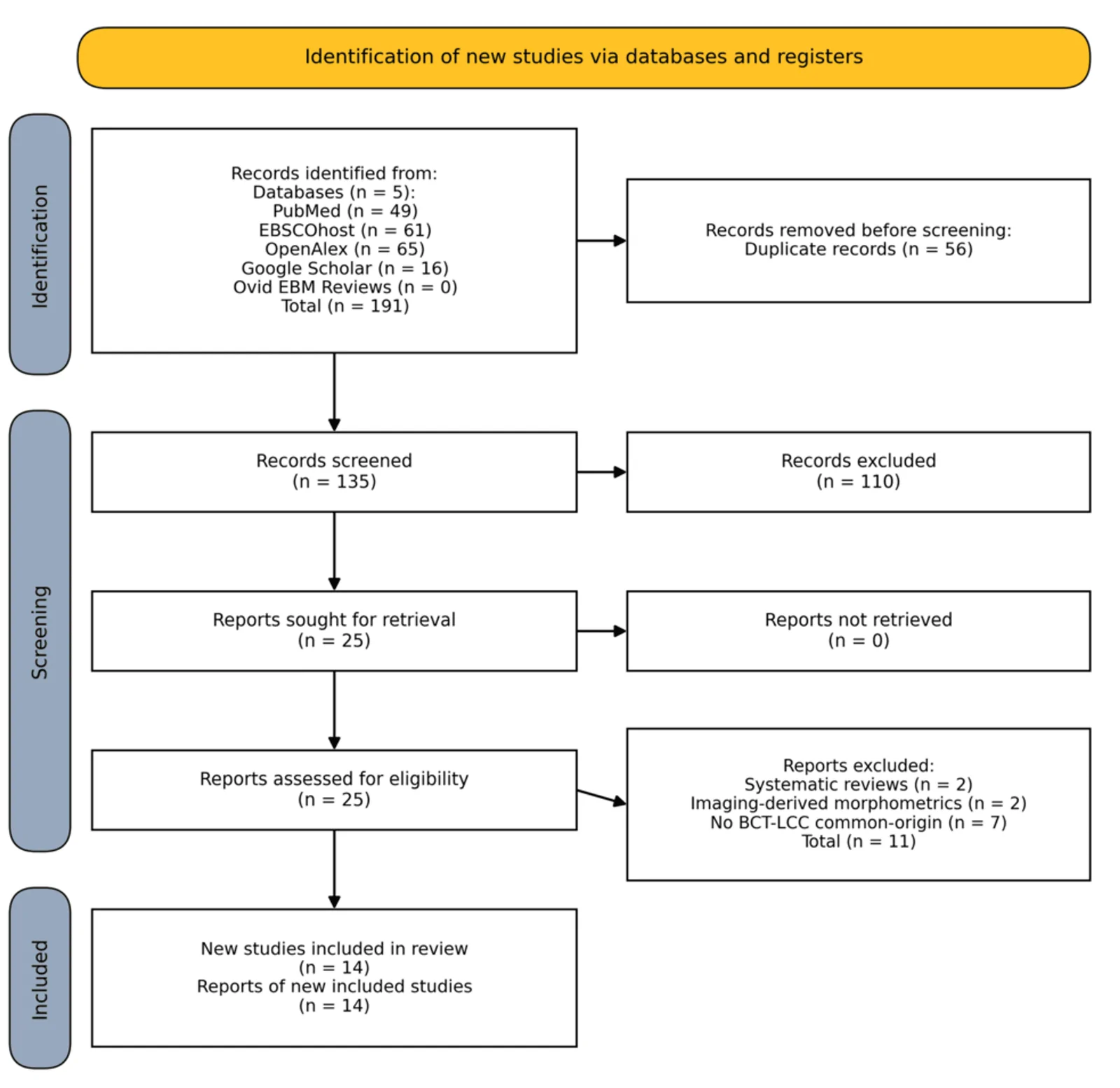
PRISMA 2020 flow diagram depicting study identification, screening, and inclusion Records were identified from five database sources and pooled for deduplication before screening. Google Scholar was entered as a database line rather than under other methods, since all sources were deduplicated together prior to screening. The diagram was generated using the PRISMA 2020 R package and Shiny application [14] PRISMA: Preferred Reporting Items for Systematic Reviews and Meta-Analyses; BCT: brachiocephalic trunk; LCC: left common carotid artery

Two publications by Natsis et al. required separate consideration. Natsis et al. 2011 is titled as a review but additionally reports primary anatomical findings from two dissected specimens with a bicarotid trunk, therefore meeting two exclusion criteria [15]. Similarly, Natsis et al. 2021 is a systematic classification review and was excluded as non-primary, consistent with its title and content [1]. These publications are unrelated apart from shared authorship and are identified distinctly under systematic reviews in the PRISMA figure to prevent conflation. Characteristics of the 14 included studies are summarized in Table 2 [8-12,16-24].

**Table 2:** Characteristics of the 14 cadaveric studies included in the systematic review ᵃThirty cadavers plus six separate hearts; distances were measured in the 30 cadavers only. ᵇTwelve percent of specimens showed two lumenal openings across three subtypes; the bovine-type configuration accounted for 7.0% of the total series. ᶜSix additional cadavers with branching variants were excluded from measurement but included in the reported percentages, which resolve against a combined total of 81. ᵈCounts (44/4/2) cited; the study's figure and discussion report 90%/8%/2% n denotes cadavers, isolated arches, or hearts examined, as reported by each source. The bovine data reported column distinguishes studies reporting morphometry specific to the bovine-type configuration from those documenting descriptive observations without measurements. Across all included studies, 486 arches or specimens were examined, of which 48 demonstrated an eligible bovine-type configuration NR: not reported

Study	Design	N	Sex (M/F)	Age (years)	Population	Bovine, n (%)	Bovine data reported	JBI overall
Allardyce et al., 2023 [16]	Cross-sectional	9	3/6	82.7 ± 10.4	West of Scotland	1 (11.1)	Configuration only	Low
Alsaif and Ramadan, 2010 [17]	Cross-sectional	36^a^	NR	NR	Jeddah, Saudi Arabia	6 (16.7)	Trunk diameter	High
Babu and Sharma, 2015 [8]	Case report	1	1/0	68	Muzaffarnagar, India	1	Trunk diameter, interostial distance	Low
Gupta and Sodhi, 2005 [12]	Cross-sectional	100	NR	40–70	Chandigarh, India	7 (7.0)^b^	Ostial diameter, interostial distance	Moderate
Junagade and Mukherjee, 2015 [18]	Cross-sectional	35	NR	50–70	Navi Mumbai, India	3 (8.6)	Trunk diameter	Low
Kodama et al., 1989 [11]	Case report	1	0/1	92	Fukuoka, Japan	1	Trunk diameter, trunk length	Low
Mohor, 2014 [19]	Cross-sectional (fresh)	75	46/29	18–74	Sibiu, Romania	2 (2.5)^c^	Configuration only	Moderate
O'Malley et al., 2018 [9]	Cross-sectional	24	9/15	NR	Dublin, Ireland	3 (12.5)	Carotid take-off length	Low
Ranjan et al., 2026 [20]	Cross-sectional	50	NR	30–70	Eastern Uttar Pradesh, India	4 (8)^d^	Configuration only	High
Rojas et al., 2017 [10]	Cross-sectional	35	NR	NR	Bogotá, Colombia	4 (11.4)	Trunk diameter	Moderate
Shin et al., 2008 [21]	Cross-sectional	25	17/8	63 (28–87)	South Korea	2 (8)	Configuration only	Moderate
Tito et al., 2023 [22]	Cross-sectional	58	57/1	NR	Uganda	7 (12)	Configuration only	Moderate
Turgut et al., 2001 [23]	Case report	1	1/0	70	Ankara, Turkey	1	Variant carotid diameter	Low
Vijayalakshmi et al., 2025 [24]	Cross-sectional	36	NR	NR	Visakhapatnam, India	6 (16.7)	Configuration only	Low

Study designs included 11 cross-sectional cadaveric series and three single-specimen case reports, with sample sizes ranging from one to 100 cadavers. Across all included studies, 486 arches or specimens were examined, of which 48 demonstrated an eligible bovine-type configuration. Eight included studies reported some measurement(s) specific to the BAA configuration, although only four of these provided values interpretable against a stated measurement convention [8,9,12,18]. This limited yield reflects the paucity of BAA-specific morphometric data rather than an inadequacy of the search strategy. The remaining six studies documented an eligible BAA configuration without reporting bovine-specific dimensions, thus informing prevalence estimates without providing morphometric comparators. This distribution is addressed further in the Critical Appraisal section.

Critical appraisal

Included studies were appraised with design-specific Joanna Briggs Institute (JBI) critical-appraisal instruments. The Checklist for Case Reports (eight items, C1 to C8) was applied to the three single-specimen reports: Babu and Sharma [8], Kodama et al. [11], and Turgut et al. [23], whereas the Checklist for Analytical Cross-Sectional Studies (domains D1 to D8) was employed for the 11 cadaveric series [25]. Complete summary risk-of-bias assessments are presented in Figures 5-6 [26].

**Figure 5:**
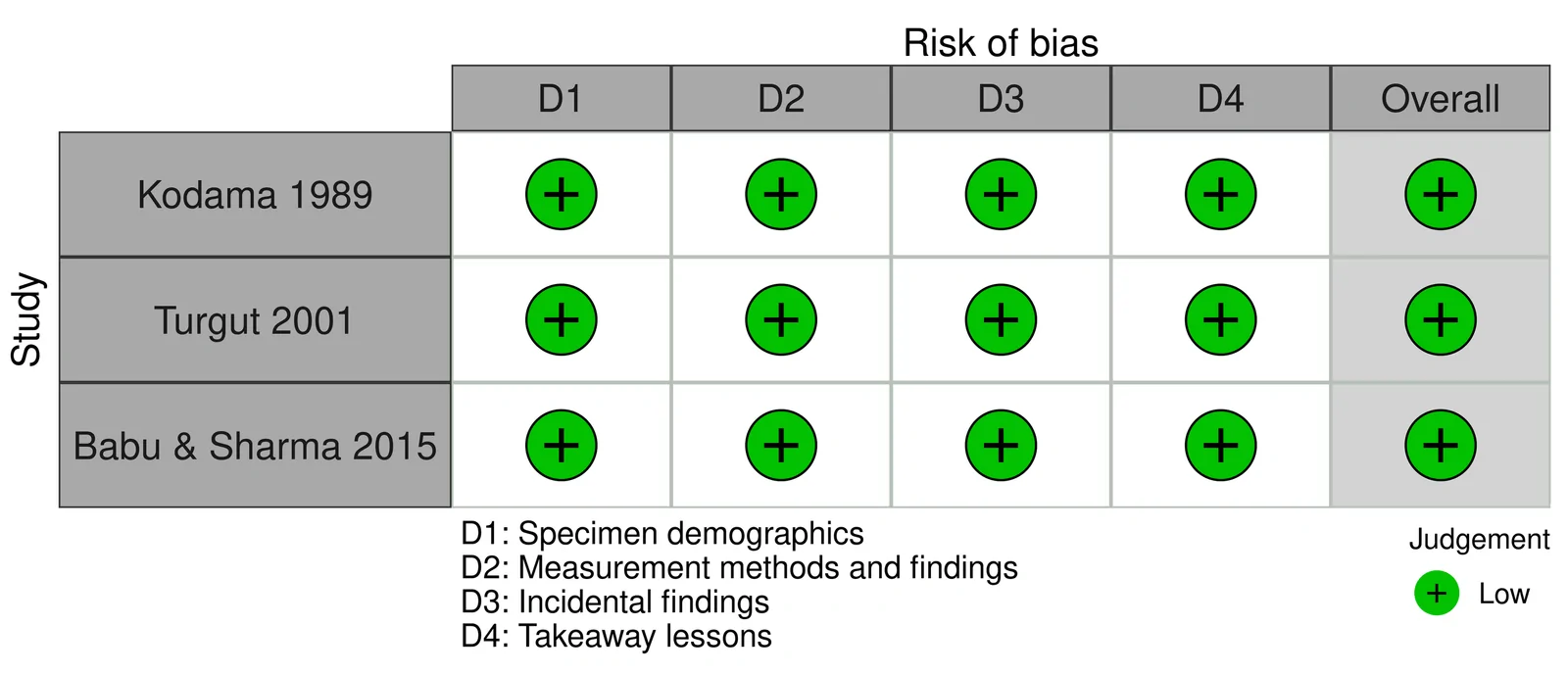
Risk-of-bias assessment of the three included single-specimen case reports Studies were appraised using the Joanna Briggs Institute Critical Appraisal Checklist for Case Reports: Kodama et al., 1989 [11], Turgut et al., 2001 [23], and Babu and Sharma, 2015 [8]. Item labels were adapted to cadaveric material ("patient" to "specimen"; "diagnostic tests or assessment methods and results" to "measurement methods and findings"; "adverse events" to "incidental findings"); the underlying appraisal criteria and response options were unchanged. Four checklist items presupposing a living patient, namely history and timeline, clinical condition on presentation, intervention, and post-intervention condition, were scored as not applicable for all three reports and are omitted from the plot; the four assessable items are shown as D1 to D4. Figure generated using robvis [26]

**Figure 6:**
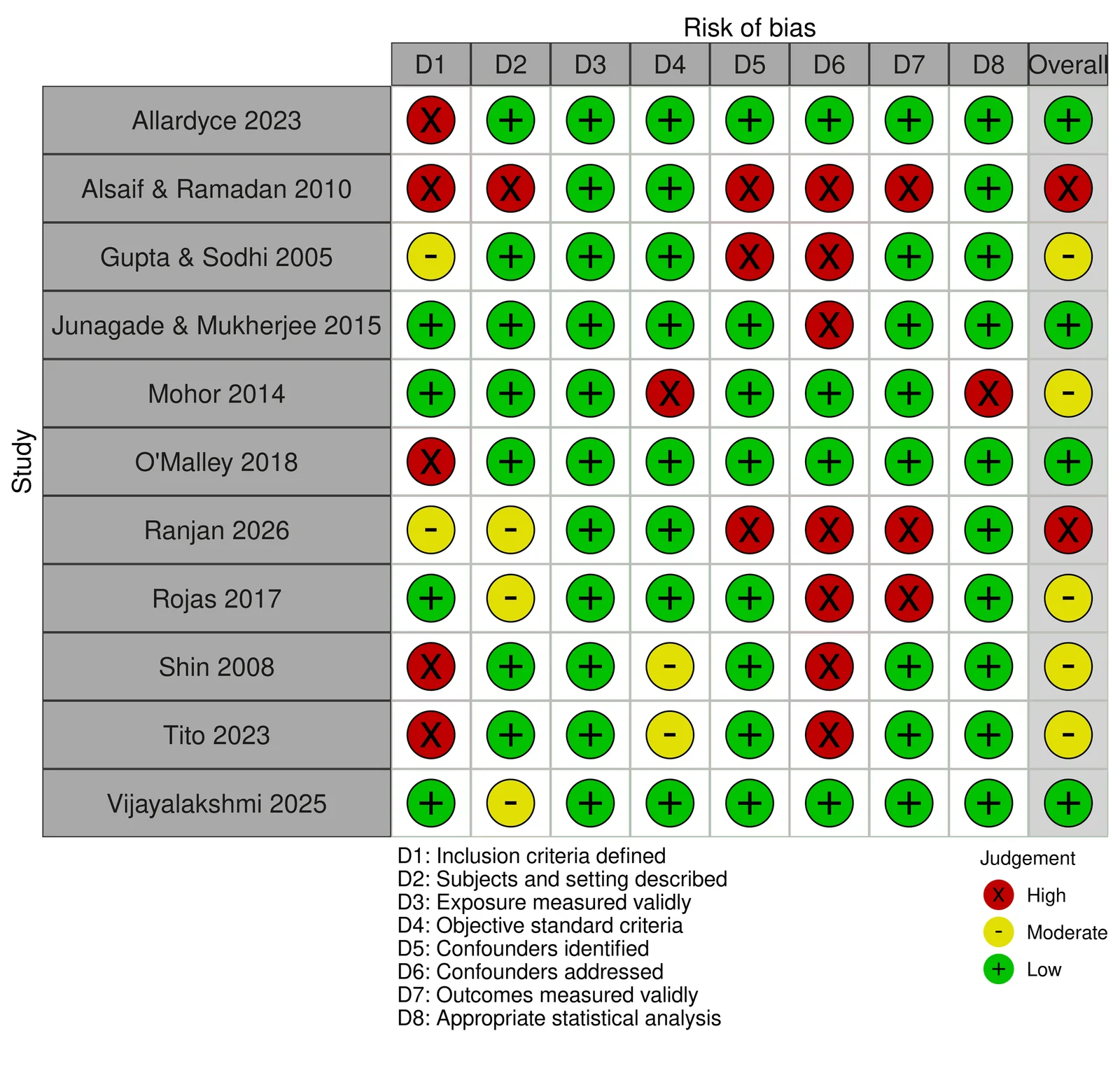
Risk-of-bias assessment of the 11 included analytical cross-sectional studies Studies were appraised using the Joanna Briggs Institute Critical Appraisal Checklist for Analytical Cross-Sectional Studies: Allardyce et al., 2023 [16], Alsaif and Ramadan 2010 [17], Gupta and Sodhi 2005 [12], Junagade and Mukherjee 2015 [18], Mohor 2014 [19], O'Malley et al., 2018 [9], Ranjan et al., 2026 [20], Rojas et al., 2017 [10], Shin et al., 2008 [21], Tito et al., 2023 [22], and Vijayalakshmi et al., 2025 [24]. Domains D1 to D8 correspond to the checklist items listed beneath the plot. Judgments were mapped to three levels, with moderate indicating partial rather than absent reporting in the relevant domain. Figure generated using robvis [26]

JBI item labels were adapted for cadaveric specimens while preserving the original criteria and response options. Specifically, “patient” was replaced with “specimen”; “diagnostic tests or assessment methods and results” with “measurement methods and findings”; and “adverse events” with “incidental findings.” Of the eight case-report items, C2, C3, C5, and C6 presuppose a living patient’s history, clinical presentation, intervention, or post-intervention course. These items were therefore designated not applicable for all three reports, leaving C1, C4, C7, and C8 as the assessable domains.

Checklist responses for the cross-sectional instrument were mapped to a three-level risk judgment: low, moderate, and high. Moderate risk was assigned when studies reported partial rather than absent methodological detail, such as specifying an age range without exclusion criteria or depicting a measuring instrument without naming it or stating its precision. Because full texts were obtained for every included study, each domain could be resolved to a substantive judgment, and failure to report was treated as absence of the methodological safeguard rather than as ambiguity. This mapping was applied uniformly, and the underlying checklist items and response options were unchanged.

Because formalin-related tissue shrinkage affects cadaveric specimens systematically and cannot be retrospectively corrected, fixation was not considered a between-study source of differential bias. Generic a priori exclusions, such as prior thoracic surgery or trauma, were credited under study-population definition rather than confounder management. Conversely, measures addressing potentially variable sources of bias, including sex- or age-stratified analyses, repeated measurements, or explicit bias-reduction procedures, were credited when reported.

Among the eleven cross-sectional studies, four were rated overall low risk of bias, five moderate, and two high. All three case reports were rated low risk [8,11,23]. Reporting of measurement methodology showed considerable spread. Mohor named neither the instrument nor the precision and was rated high in this domain [19], while Shin et al. and Tito et al. depicted a measuring device in their figures without naming it or stating its precision, and were rated moderate [21,22]. Alsaif and Ramadan, Rojas et al., and Ranjan et al. either did not state whether diameters were internal or external, or reported values that could not be reconciled internally, and were rated high for outcome validity [10,17,20]. This recurrent reporting deficiency intrinsically generates the measurement-convention problem addressed later in the Discussion.

Explicit eligibility criteria were stated in four of the eleven cross-sectional studies. Two further studies specified an age range without stating exclusion criteria and were rated moderate in this domain, while the remaining five stated no eligibility criteria and were rated high. A comparable gradation applied to sample description: three studies characterized their setting and specimen type but omitted demographic detail or left sample attrition unexplained, and were rated moderate, whereas one study reported neither age nor sex and pooled specimens of mixed provenance. These patterns recurred across decades and populations, independently corroborating the recurrent measurement-convention limitations discussed below.

Bovine trunk caliber across the literature

The brachiocephalic trunk of the present specimen measured 18.73 mm externally and 17.1 mm luminally, with both values confirmed by repeat direct caliper assessment. Few cadaveric BAA-specific comparators exist, but the interpretable values cluster narrowly (Table 3). Babu and Sharma reported an external diameter of 18.84 mm and an ostial diameter of 17.35 mm [8]. Junagade and Mukherjee reported mean external and luminal diameters of 18.39 mm and 16.85 mm across three specimens, respectively [18]. The present external diameter differs from these two external values by 0.11 mm and 0.34 mm, respectively, and the present luminal diameter differs from the ostial diameter of Babu and Sharma by 0.25 mm (Table 3). Gupta and Sodhi measured the common brachiocephalic-left common carotid ostium on the opened endoluminal surface [12].

**Table 3:** Bovine-specific morphometry reported across the cadaveric literature, separated by measurement convention ᵃConvention not stated in the source; value tabulated in this column rather than assigned to external or luminal. ᵇImaging-derived; contrast-enhanced CT delineates the lumen. ᶜOstial diameter on two axes, maximum anteroposterior (AP) and side-to-side (SS), reflecting oval ostial geometry in 90% of specimens; not equivalent to a single-axis vessel diameter. ᵈMeasured from a paraffin-wax reference plane along the superior arch wall to the apex of the internal junction between the brachiocephalic and left common carotid lumens; the same source separately notes a 7.0 mm external ascent before division. Kodama also reported an aortic arch diameter of 34.5 mm in a 92-year-old female specimen [11] Diameters are reported separately as external and luminal values, which differ by approximately 1.5 mm across sources reporting both. The interostial interval is reported as measured on the intact external surface or endoluminally after opening the arch; for an interval bounded by two ostia, the endoluminal value exceeds the external value because it incorporates the mural thickness of each bounding vessel. Studies are ordered by number of bovine parameters reported. Turgut et al. report a variant left common carotid diameter of 13.0 mm and a brachiocephalic trunk length of 42.0 mm to the terminal bifurcation, neither comparable to the tabulated parameters, and are therefore not represented [23] CT: computed tomography

Study	Type	Trunk diameter, external (mm)	Trunk diameter, luminal (mm)	Trunk diameter, unstated convention (mm)	Trunk length/carotid take-off (mm)	Interostial interval (mm)
Present case	Cadaveric case	18.73	17.1	—	8.01	7.18 (externally) and 8.5 (endoluminally)
Babu and Sharma, 2015 [8]	Cadaveric case	18.84	17.35	—	6.0	12.91 (endoluminally)
Junagade and Mukherjee, 2015 [18]	Series (3 bovine)	18.39	16.85	—	9.33 (8–10)	—
Gupta and Sodhi, 2005 [12]	Series (7 bovine)	—	17.5 ± 1.3 AP/15.5 ± 1.3 SS^c^	—	—	9.6 ± 1.5 (4–12); (endoluminally)
Kodama et al., 1989 [11]	Cadaveric case	—	—	17.2^a^	11.0^d^	—
Alsaif and Ramadan, 2010 [17]	Series (6 bovine)	—	—	30.33 ± 5.16^a^	15.00 ± 5.86 (10–26)	—
O'Malley et al., 2018 [9]	Series (3 bovine)	—	—	—	17.3 ± 8.6 (10.0–26.8)	—
Rojas et al., 2017 [10]	Series (4 bovine)	—	—	10.1 ± 1.37^a^	—	—
Mertens et al., 2020^b ^[27]	Imaging series	—	—	—	—	12.4 ± 5.9 (2–27); (endoluminal imaging)

In the bovine subgroup of their series, they reported a maximum anteroposterior diameter of 17.5 ± 1.3 mm and a maximum transverse diameter of 15.5 ± 1.3 mm [12]. These two axes bracket the present luminal diameter and reflect the oval ostial geometry described in 90% of their specimens, a two-axis convention that is not directly equivalent to a single-axis vessel diameter. Kodama et al. reported a common-trunk diameter of 17.2 mm at its origin and a longitudinal length of 11.0 mm, confirming a complete single endoluminal ostium; however, the diameter convention was unspecified [11]. Turgut described a left common carotid artery arising from the brachiocephalic trunk, with a diameter of 13.0 mm [23]. In this configuration, the shared segment corresponds to the brachiocephalic trunk; thus, the reported measurement characterizes the left common carotid branch rather than the shared trunk, precluding direct comparison, and that study is therefore not tabulated.

Two reports lie outside the principal cluster. Alsaif and Ramadan reported a common-trunk diameter of 30.33 ± 5.16 mm across six specimens [17], whereas Rojas et al. reported a common-trunk diameter of 10.1 ± 1.37 mm across four samples [10]. Neither study specified whether diameters were obtained externally or endoluminally. The Rojas value is anatomically difficult to reconcile because it only marginally exceeds that study’s conventional brachiocephalic-trunk mean of 9.95 mm, despite the larger perfusion territory served by a shared trunk. In contrast, diameters throughout the Alsaif and Ramadan series were systematically greater, suggesting a differing measurement convention rather than true pathological enlargement.

Normal-arch comparators further support an increased expected caliber of the shared trunk. Zhao et al., in a cadaveric study of the brachiocephalic trunk-tracheal relationship, reported a male external brachiocephalic-trunk diameter of 14.24 ± 2.37 mm using an explicitly stated convention, ~4.5 mm below the bovine cluster [28]. Shin et al. reported a luminal mean of 18.3 ± 7.0 mm and a maximum of 31.5 mm in a cohort reflecting two BAA specimens [21], suggesting a potential BAA contribution to the statistical upper tail range. Accordingly, the large-caliber conduit observed in the present specimen is commensurate with the expected combined carotid outflow, an interpretation supported by the absence of aneurysmal dilatation, mural thrombus, or calcification.

Aortic arch caliber was likewise within the spectrum of normal anatomical variation. The present mid-arch external diameter of 25.18 mm was 16.1% below the 3.0 cm male mean reported by Rylski et al. [29]. Of note, that value was measured by contrast-enhanced computed tomography from the outer aortic wall at the mid-arch level, providing the closest available correspondence in measurement convention and anatomical plane. The reference cohort presented a median age of 57 years, with an expected increase in male arch diameter by ~2% per decade [29], potentially placing the present measurement modestly further below the age-matched expected mean. Formalin fixation may contribute to measurement discrepancies via tissue shrinkage, compounded by the absence of hemodynamic perfusion in cadaveric specimens compared with in vivo imaging (addressed in study limitations). Comparable external arch diameters are reported by Zhao et al. [28], Junagade and Mukherjee [18], and Vijayalakshmi [24] (Table 4); Ranjan and colleagues [20] report values substantially below this range and are not used for comparison, consistent with the non-reconcilable morphometry noted for that study in Table 2.

**Table 4:** Aortic arch diameter in the present specimen and comparator series ^a^Diameter calculated from the cross-sectional aortic surface in reference to the outer wall, at a plane in the middle of the arch segment, with additional planes immediately proximal to the brachiocephalic orifice and immediately distal to the left subclavian orifice, corresponding to the three planes reported for the present specimen The present specimen is the only cadaveric source reporting a three-point proximal-to-distal profile. Rylski et al. report the same three arch planes measured to the outer wall on an imaging comparator via contrast-enhanced computed tomography; that cohort had a median age of 57 years and is not age-matched to the present specimen [29]. Kodama et al. [11] and Rojas et al. [10] report arch diameters without stating a measurement convention. Gupta and Sodhi are omitted, as they report the luminal surface only [12]. Ranjan et al. reported arch diameters of 18.67 mm vertical and 15.71 mm transverse, values not reconcilable with the remaining literature, and are excluded from this comparison [20]

Source	Arch diameter (mm)	Specimens or cohort	Convention
Present case	28.68 proximal/25.18 mid/24.15 distal	Male, late seventies	External (caliper, intact vessel)
Rylski et al., 2014 [29]	30 (M)/27 (F)	195 adults, median age 57, contrast CT	External (outer wall)^a^
Zhao et al., 2023 [28]	31.00 ± 5.17 (M)/27.77 ± 5.21 (F)	91 cadavers	External (stated)
Junagade and Mukherjee, 2015 [18]	26.44 ± 4.61 origin/19.22 ± 3.43 termination	35 cadavers	External
Vijayalakshmi et al., 2025 [24]	25 ± 3	36 cadavers	External
Rojas et al., 2017 [10]	23.4 ± 3.36	35 cadavers	Not stated
Kodama et al., 1989 [11]	34.5	Female, 92 years	Not stated

Interbranch distance and the problem of measurement convention

The distinction between a true common trunk and an apparently confluent origin was recognized as a source of between-study variation as early as 1989, when Kodama and colleagues reported that approximately half of apparently confluent origins prove not to be true common trunks on endoluminal inspection [11]. In the present case, the single ostial interval between the trunk and the left subclavian artery measured 7.18 mm on the intact external surface and 8.5 mm endoluminally following an anterior arteriotomy (Figure 7). The greater endoluminal interval reflects mural thickness at the bounding vessel origins; the observed 1.32 mm difference accords with the 1.63 mm external-to-endoluminal diameter difference of the brachiocephalic trunk in the same specimen.

Two prior cadaveric reports provide comparable measurements, both made on the opened endoluminal surface [8,12]. Babu and Sharma, after exposing a window in the arch wall, reported 12.91 mm between the two ostia on the exposed endoluminal surface in a structurally identical two-branch arch, though their second ostium was a vertebro-subclavian trunk rather than a native left subclavian artery [8]. Gupta and Sodhi reported a mean transverse distance of 9.6 ± 1.5 mm (range 4 to 12) from the inner margin of the common brachiocephalic-left common carotid ostium to that of the left subclavian ostium in the bovine subgroup of a 100-specimen series [12]. The present endoluminal interval of 8.5 mm is 34.2% below the Babu and Sharma value and 11.5% below the Gupta and Sodhi mean, while remaining within the lower segment of that study's reported range.

**Figure 7:**
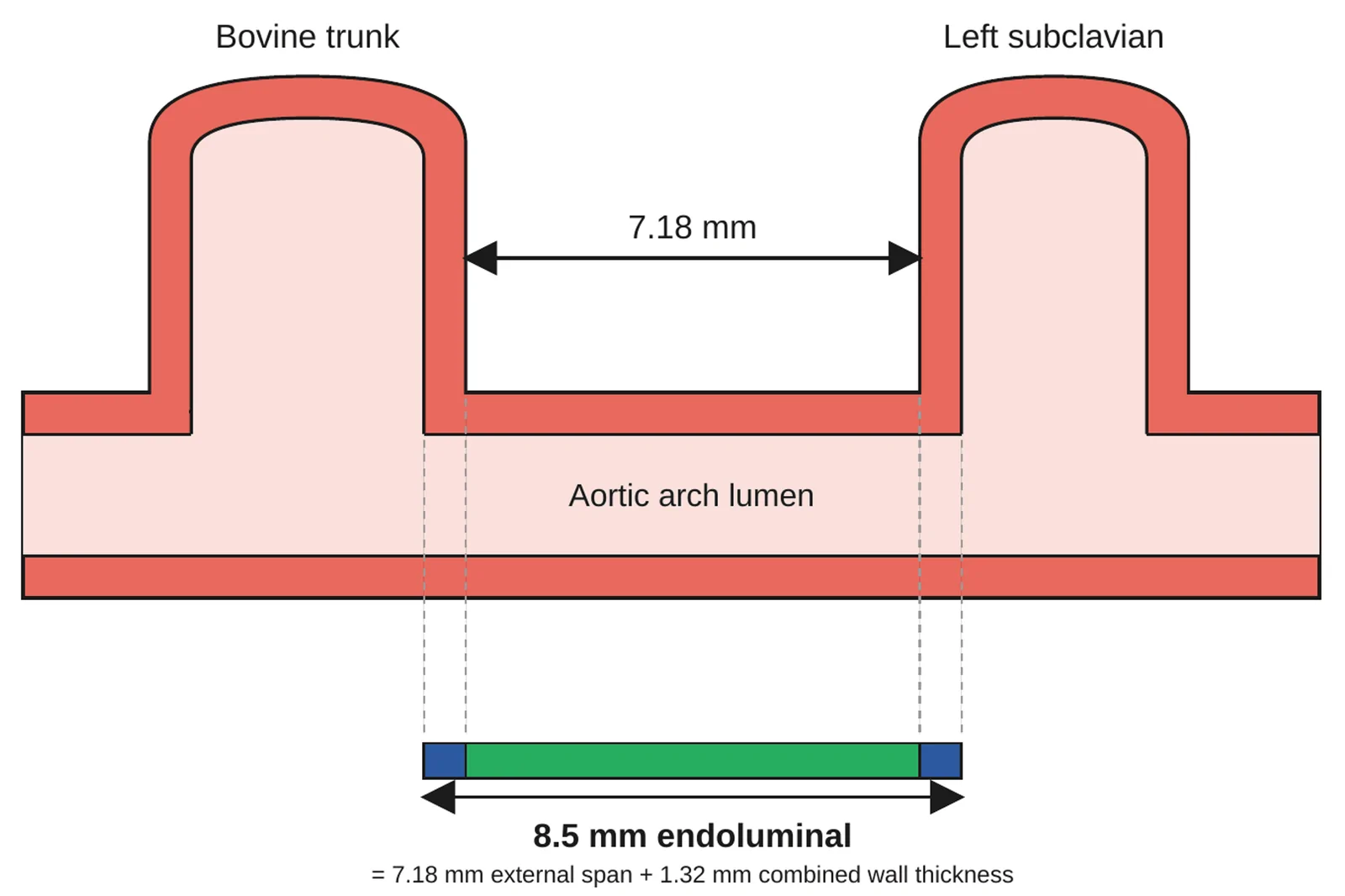
Interpretive geometric model of the external and endoluminal interostial intervals Schematic cross-section through the aortic arch wall at the level of the two branch origins, depicting the bovine-type trunk and left subclavian artery with their respective mural thickness. The external interval spans the outer walls of the two vessels at their point of closest apposition on the intact specimen, excluding both walls from the measured span. In contrast, the endoluminal interval spans the two ostial rims following arteriotomy, each rim recessed from its vessel's outer footprint by one mural thickness, such that both walls fall within the measured span. Under this model, the larger endoluminal value is attributable to the combined mural thickness of the bounding vessels, inherently reflected in the observed 1.32 mm differential between the two interostial measurements. Blue segments represent the combined mural thickness of the two bounding vessels (1.32 mm total), shown as equal segments for schematic clarity. Original schematic created by the authors using BioRender.

The closest same-landmark comparator is imaging-derived. Mertens and colleagues, in a series of bovine-type arches assessed by computed tomography, reported a trunk-to-left subclavian distance of 12.4 ± 5.9 mm, median 13 mm, range 2 to 27 mm [27]. The present endoluminal value is 31.5% below that mean, though it lies approximately 0.66 standard deviations below it and well within the reported range, indicating a compressed interval. The comparison remains directional, since it sets fixed-tissue caliper measurement against imaging of a perfused vessel.

A distinct parameter deserves separation. The distance from the trunk's aortic origin to the emergence of the left common carotid artery, the carotid take-off length, measured 8.01 mm. Five prior cadaveric sources report this parameter, several under the label common-trunk length: Babu and Sharma 6.0 mm [8], Junagade and Mukherjee 9.33 mm (range 8 to 10) [18], Kodama 11.0 mm [11], Alsaif and Ramadan 15.00 ± 5.86 mm (range 10 to 26) [17], and O'Malley and colleagues 17.3 ± 8.6 mm (range 10.0 to 26.8) [9]. Alsaif and Ramadan [17] define this measurement explicitly as extending from the trunk's origin to its bifurcation, and Junagade and Mukherjee [18] compare their value directly against it, confirming parameter equivalence. The present value of 8.01 mm falls at the lower end of this distribution. Compression is therefore demonstrable for the ostial interval, with a comparably reduced interspecimen take-off length. The two measurements are nonetheless frequently conflated despite describing perpendicular quantities: the ostial interval runs along the arch between adjacent ostia, while the take-off length runs proximally along the trunk to the carotid branch point.

The scarcity of usable comparators reflects a structural fact. A bovine two-branch arch has exactly one inter-ostial interval, while a normal three-branch arch has two, separated by the left common carotid ostium. The normal-arch brachiocephalic-to-left subclavian span therefore measures a different anatomical distance altogether, conflating carotid relocation with genuine compression. Comparisons here are accordingly restricted to bovine specimens. Among normal arches, reported interbranch distances exhibit variation exceeding that attributable to anatomical heterogeneity alone (Table 5). Brachiocephalic-to-left common carotid intervals range from 2.97 to 18.2 mm, representing a greater than sixfold difference. Three studies provide sufficiently explicit methodological descriptions to permit reconstruction of their distinct landmark conventions. Mohor defined and illustrated the measurement as the gap between adjacent vessel walls, reporting 2.97 mm in males [19]. Allardyce and colleagues measured interbranch spacing by pinning each vessel at its midpoint, reporting 18.2 mm [16]. Gupta and Sodhi measured the exposed endoluminal interval between the inner margins of adjacent ostia, reporting 5.0 ± 1.2 mm [12].

**Table 5:** Interbranch distances reported in normal three-branch arches, showing the effect of measurement convention ^a^Derived as S1 + LCC diameter + S2 (2.97 + 7.15 + 6.89) for male specimens. ^b^Cited for context. ^c^Brachiocephalic trunk to right subclavian artery, a different parameter. ^d^Derived as BCT–LCC + LCC ostial width + LCC–LSA (5.0 + 5.2 + 8.0), inner margin to inner margin. ^e^Normalized by adding the radius of each bounding ostium, taken from that study's reported maximum side-to-side ostial diameters (BC 6.9, LCC 5.2, LSA 5.8 mm). The derivation assumes the reported transverse inter-ostial distance and the side-to-side ostial diameter share a common axis, which the source implies but does not state explicitly. Normalized values are reported to the precision of the inputs from which they were derived; the Gupta and Sodhi transformations yield 11.05 and 24.55 mm, reported here as 11.1 and 24.6 mm Before accounting for differing landmark conventions, reported values span more than sixfold. Three studies state their convention explicitly, and the three conventions differ: external edge-to-edge, midpoint-to-midpoint, and endoluminal inner-margin-to-inner-margin. Each reported interval was transformed to an estimated midpoint-to-midpoint distance by adding half the diameter of each bounding vessel or ostium. Normalizing the Mohor [19] edge-to-edge values brings the brachiocephalic-to-left subclavian distance to an estimated 27.24 mm, closely matching the 27.3 mm reported independently by Gautam et al. [30]; the same transformation applied to Gupta and Sodhi yields an estimated 24.6 mm [12]. Estimated values are illustrative and should not be interpreted as directly measured anatomical distances. Studies not stating a convention cannot be normalized BCT: brachiocephalic trunk; LCC: left common carotid artery; LSA: left subclavian artery

Study	BCT-LCC (mm)	BCT-LSA (mm)	Stated convention	Normalized to midpoint
Mohor, 2014 [19]	2.97 (M)/3.06 (F)	17.01^a^	Edge-to-edge (defined and illustrated)	BCT–LCC 12.16; BCT–LSA 27.24
Allardyce et al., 2023 [16]	18.2 ± 3.3	37.8 ± 6.3	Midpoint-to-midpoint (stated)	Already midpoint
Gupta and Sodhi 2005 [12]	5.0 ± 1.2 transverse/3.0 ± 1.2 vertical	18.2^d^	Endoluminal, inner margin to inner margin (stated)	BCT–LCC 11.1; BCT–LSA 24.6^e^
Gautam et al., 2026^b ^[30]	11.4 (M)/10.6 (F)	27.3 (M)/25.8 (F)	Not stated	—
O'Malley et al., 2018 [9]	—	34.1 ± 8.8^c^	Pin at vessel origin	—
Vijayalakshmi et al., 2025 [24]	—	48	Not stated	Not derivable

Normalization of these values to a common midpoint-to-midpoint landmark framework substantially reduces the apparent interstudy discrepancy. Each reported interval was transformed by adding half the diameter of each bounding vessel or ostium, such that $D_{mid} = D_{\mathrm{reported}} + \frac{D_1 + D_2}{2}$, where D_mid_ is the estimated midpoint-to-midpoint distance, D_reported_ is the published edge-to-edge or inner-margin distance, and D₁ and D₂ are the diameters of the two bounding vessels or ostia. This transformation assumes approximately circular cross-sections and correspondence between the reported diameter and distance axes, neither of which was described in the source studies. Converting Mohor's measurement by incorporating the radii of the intervening vessels yields an estimated brachiocephalic-to-left common carotid interval of 12.16 mm; the corresponding brachiocephalic-to-left subclavian span is 27.24 mm, closely approximating the independently reported 27.3-mm value of Gautam et al. [30].

Applying the same normalization to Gupta and Sodhi yields $D_{mid} = 5.0 + \frac{6.9 + 5.2}{2} = 11.05 \; \mathrm{mm}$, ​where 6.9 mm and 5.2 mm are the maximum side-to-side ostial diameters of the brachiocephalic and left common carotid ostia, respectively. Similarly, the corresponding brachiocephalic-to-left subclavian span is 24.6 mm, both reported to one decimal place for source precision. Thus, three series employing distinct measurement conventions demonstrate close concordance after landmark harmonization, despite an unadjusted reported range of 2.97 to 48 mm. Measurement convention likely accounts for much of this apparent interstudy variability.

Several studies report distances without stating a convention. Vijayalakshmi and colleagues rigorously recorded three repeated measurements by two independent observers and reported a brachiocephalic-to-left subclavian distance of 4.8 cm, defined as the distance between the origins of the two vessels [24]. Whether this represents an edge-to-edge, ostium-center, or along-curvature measurement is not specified; the value therefore cannot be positioned relative to the studies above. Measurement precision and landmark specification are separable, and both are required for values to be pooled across studies. For this reason, the present report states its own conventions explicitly in the Case Presentation and provides both external and endoluminal values throughout.

Hemodynamic and remodeling considerations

Three distinct branches arise at regular intervals in standard aortic arch anatomy, promoting more symmetric flow distribution and optimal shear stress dispersion [31]. The aortic arch may be conceptualized as a branching vascular network in which flow partitioning depends on vessel caliber and interbranch spacing. The present specimen only documents branching geometry and vessel caliber but does not measure flow, pressure, or wall shear stress. However, idealized flow relationships may offer a conceptual and useful geometrical lens. Poiseuille's relationship predicts that modest caliber changes produce disproportionate flow effects, as resistance varies inversely with the fourth power of radius. Wall shear stress likewise scales inversely with the cube of radius; thus, an enlarged brachiocephalic trunk carrying combined carotid outflow would be expected to alter proximal shear conditions. Accordingly, the large-caliber brachiocephalic trunk and compressed branching pattern in this specimen likely modify local flow and wall shear stress.

Shalhub and colleagues demonstrated elevated peak systolic wall shear stress along the inner curvature of the BAA compared with conventional arch anatomy, using 4D flow MRI in patients with type B aortic dissection [5]. Idealized computational fluid dynamics and in vitro models similarly reproduce geometry-dependent hemodynamic signatures that vary with arch curvature and branch-vessel caliber [32]. Together, these findings offer in vivo and experimental evidence that a shared carotid origin alters regional hemodynamics. This is mechanistically relevant because elevated wall shear stress is associated with aortic wall remodeling: in ascending aortic aneurysm tissue, high-shear regions show measurable reductions in elastin content, wall thinning, and decreased vascular smooth muscle cell density [33]. Over time, such focal shear stress amplification may promote adaptive remodeling or predispose to endothelial dysfunction and intimal injury at branch points, even in the absence of overt macroscopic pathology [34]. The absence of aneurysmal dilatation, mural thrombus, or calcification in the present specimen does not exclude these mechanisms, since fixed tissue captures only a single static time point and cannot demonstrate whether focal shear amplification was present during life.

Clinical and procedural implications

Clinically, the BAA variant carries implications for open, hybrid, and endovascular interventions. During aortic arch repair, stent-graft placement, or carotid-subclavian bypass, a shortened trunk and shallow take-off angle can increase technical difficulty and the risk of malperfusion or embolization [6,7]. Two contemporary series provide adjusted, configuration-specific estimates. Among 2,775 patients undergoing transcatheter aortic valve replacement, bovine-type arch anatomy was independently associated with periprocedural stroke (3.6% versus 1.7%; adjusted odds ratio 2.16, 95% CI 1.22-3.83) and with stroke over a median 2.7-year follow-up (10.5% versus 5.0%; adjusted hazard ratio 2.11, 95% CI 1.51-2.93; P<0.001) [35]. In a multicenter series of 963 patients undergoing thoracic endovascular repair for Stanford type B dissection, early stroke occurred in 8.0% of those with a bovine-type arch versus 3.0% with standard arch anatomy (adjusted odds ratio 2.01, 95% CI 1.49-2.68; P=0.012) [36]. The narrowed supra-aortic corridor implicated in this elevated risk is quantified directly in the present specimen: an endoluminal ostial interval 34.2% below the closest comparable cadaveric value [8] and 11.5% below the only cadaveric series reporting this parameter [12], with a carotid take-off length falling at the lower end of the reported cadaveric range. The interostial finding describes a working corridor for endovascular guidewire and catheter manipulation narrower than existing bovine comparators suggest.

This report adds specimen-level morphometric detail to the limited cadaveric literature on bovine-type aortic arch variants and emphasizes the value of preoperative arch characterization for procedural planning. Cadaveric dissection remains uniquely valuable for anatomical resolution not readily achievable by imaging alone, particularly for endoluminal ostial relationships that require an anterior arteriotomy. Progress in this field depends less on additional prevalence data than on consistent reporting. As shown above, values can be compared across studies only after reconstruction of the underlying landmark convention, permitting direct and accurate comparison. Measurement ambiguity could be minimized by specifying whether measurements are external or endoluminal, defining the landmarks bounding each interval, and reporting the ostial interval and carotid take-off length separately, as these represent perpendicular quantities that are frequently conflated.

Several directions follow. Paired cadaveric and imaging measurements of the same specimen would quantify the offset between fixed-tissue and in vivo values. Computational modeling built from BAA geometry, including the compressed configuration described here, could test whether ostial spacing and take-off length independently predict wall shear stress. Registry-based analyses correlating preoperative arch morphometry with procedural outcomes could define prognostically relevant thresholds for device selection and vascular access strategy. Until such data exist, the case for preoperative arch characterization rests on anatomical plausibility rather than demonstrated outcome benefit.

Bovine arch and aortic dissections

Beyond the procedural considerations above, the bovine-type arch has been linked to aortic dissection itself, which provides a direct route from the branching geometry described here to a concrete clinical concern. The shear-mediated remodeling and intimal-injury mechanisms offer a plausible substrate, and the reported association spans both dissection types. In acute type A dissection, Dumfarth et al. found that among 157 operated patients, a bovine arch localized the primary intimal entry tear to the aortic arch far more often than standard anatomy (59.1% versus 13.3%; odds ratio 14.79, 95% CI 4.54-48.13) and was an independent risk factor for postoperative neurologic injury (odds ratio 4.9, 95% CI 1.635-14.734) [37].

The same series found arch angulation to be more acute in patients whose entry tear was located in the arch (76.7° versus 85.9°; p = 0.024), consistent with a geometry-mediated mechanism [37]. In a larger DeBakey type I cohort of 315 patients, the same group again identified the bovine arch as an independent predictor of arch entry tears (odds ratio 5.9, 95% CI 2.89-12.04) and of postoperative stroke (24.5% versus 10.2%; odds ratio 2.69, 95% CI 1.2-6.0) [38]. In type B dissection, variant arch anatomy carries elevated peak systolic wall shear stress along the inner curvature [5], and a bovine arch is independently associated with early stroke after thoracic endovascular repair [36]. The preferential location of the entry tear at the arch in bovine anatomy is consistent with the compressed, presumptively high-shear branching geometry documented in the present specimen, and it reframes an incidentally noted bovine arch as a potentially meaningful piece of clinical context.

Study limitations

Several limitations apply to this report. First, clinical history and cause of death were unavailable for the specimen, precluding clinicopathological correlation. Additional specimen characteristics, including prior thoracic trauma and vascular history, were unavailable. Formalin-related tissue shrinkage limits all cadaveric measurements; however, its broadly comparable effect across studies renders it unlikely to be a discriminating source of interstudy morphometric variation. More fundamentally, all measurements derive from fixed tissue at a single static time point and cannot capture spatiotemporal or pulsatile hemodynamic behavior. The wall-shear-stress mechanisms discussed above derive from imaging and computational studies of physiologically perfused systems and are not directly demonstrated by this specimen. The absence of gross aneurysmal dilatation, mural thrombus, or calcification likewise does not confirm or exclude prior hemodynamic remodeling.

The systematic review carries its own limitations. Google Scholar, included as a supplementary citation-discovery source, does not support nested Boolean syntax or controlled vocabulary and ranks results through a partially personalized, time-variable relevance algorithm. Although the search strategy is fully specified, repeated searches may not yield an identical result set. Measurement conventions were unstated in most included studies, restricting direct comparison to a small subset of sources; as discussed above, this reporting deficiency constitutes a substantive finding but also limits quantitative synthesis. No pooled variance estimates or formal sensitivity analyses could inherently be performed with so few comparable series. Finally, bovine-specific morphometry was reported in a limited number of studies, with the remainder providing descriptive configurations alone, thereby precluding precise cross-study comparisons.

## Conclusions

This report integrates specimen-level morphometric characterization of a bovine-type aortic arch with a systematic review of the cadaveric literature describing this configuration. The specimen demonstrates a compressed endoluminal ostial interval, 34.2% lower than the most directly comparable cadaveric measurement, with a carotid take-off length at the lower end of the range reported across prior bovine cadaveric sources. The brachiocephalic trunk, at 18.73 mm externally and 17.1 mm luminally, falls within the limited range of directly interpretable published measurements. No gross aneurysmal dilatation, mural thrombus, or atherosclerotic calcification was observed; these findings do not establish physiologic caliber or exclude prior hemodynamic remodeling. The review found 14 eligible studies, of which eight reported some bovine-type arch-specific measurement, and only four provided values interpretable against a stated measurement convention. Two prior cadaveric reports provided potentially comparable interostial measurements, although their branch anatomy and measurement conventions differed. Where values could be compared, apparent interstudy variation was substantially influenced by measurement convention and inconsistent landmark definitions. Landmark harmonization of three independently reported series employing distinct measurement conventions yielded closely concordant estimated interbranch distances. Most included studies did not state the landmark convention on which their measurements depended.

The observed configuration could influence local shear forces at supra-aortic branch points and may restrict the working corridor for arch repair, endovascular stent-grafting, carotid stenting, and transcatheter valve procedures. Anatomical variants that appear clinically silent may therefore have potential hemodynamic and procedural relevance; however, these consequences cannot be inferred directly from fixed cadaveric specimens. Explicit reporting of measurement conventions will help accumulate and reconcile granular observations into comparable morphometric distributions. Translating these observations into reliable clinical inferences will subsequently require paired imaging-cadaveric studies, standardized morphometric protocols, and outcome-linked clinical research.

## Appendices

**Table 6:** Search strings by database We searched five databases from inception to August 21, 2026. Records from all five arms were pooled and deduplicated together before screening. Google Scholar does not support nested Boolean syntax or controlled vocabulary and returns relevance-ranked rather than fixed result sets; the first 100 results per query were screened by title as a predefined stopping rule, and the three queries together yielded 16 records. The Ovid EBM Reviews bundle comprises the Cochrane Database of Systematic Reviews, ACP Journal Club, DARE, Cochrane Clinical Answers, CENTRAL, the Cochrane Methodology Register, HTA, and NHS EED; the null yield reflects the trial- and review-focused scope

#	Database	Interface	Search string	Records
1	PubMed	NCBI, single-string entry; all fields with MeSH tags as shown	(("Aorta, Thoracic"[Mesh] OR "aortic arch"[tiab] OR "arch of aorta"[tiab] OR "aortic arch branching"[tiab]) AND ("bovine aortic arch"[tiab] OR "bovine arch"[tiab] OR "bovine variant"[tiab] OR "common origin"[tiab] OR "common trunk"[tiab] OR "bicarotid trunk"[tiab] OR "two-branched"[tiab] OR "two branch*"[tiab] OR "shared origin"[tiab] OR "brachiocephalic trunk"[tiab]) AND ("Cadaver"[Mesh] OR cadaver*[tiab] OR "Autopsy"[Mesh] OR autopsy[tiab] OR dissection[tiab] OR embalmed[tiab] OR "formalin-fixed"[tiab] OR "anatomical specimen*"[tiab]) AND (morphometr*[tiab] OR measurement*[tiab] OR diameter*[tiab] OR "inter-branch"[tiab] OR interbranch[tiab] OR "branch spacing"[tiab] OR calliper*[tiab] OR caliper*[tiab] OR angle*[tiab] OR distance*[tiab])) AND "humans"[MeSH Terms]	49
2	EBSCOhost	Advanced Search, field TX All Text	#1 "aortic arch" OR "arch of aorta" #2 "bovine arch" OR "bovine aortic arch" OR "common trunk" OR "common origin" OR "bicarotid trunk" OR "brachiocephalic trunk" #3 cadaver* OR autopsy OR dissection OR embalmed #4 morphometr* OR measurement* OR diameter* OR calliper* OR caliper* 1 AND 2 AND 3 AND 4	61
3	OpenAlex	API, title_and_abstract.search, per-page=100	(bovine arch OR "common trunk") AND (cadaver OR dissection) AND (morphometr OR diameter)	65
4	Google Scholar	Private browsing session; first 100 results per query screened by title	Query 1 "bovine aortic arch" cadaveric morphometric brachiocephalic trunk Query 2 "aortic arch" cadaver morphometry "common trunk" diameter Query 3 bovine arch cadaveric dissection inter-branch distance measurement Queries screened independently; records pooled	16
5	Ovid EBM Reviews	All EBM Reviews bundle; numbered lines in search history	#1 aortic arch.ti,ab. OR arch of aorta.ti,ab. #2 bovine arch.ti,ab. OR bovine aortic arch.ti,ab. OR common trunk.ti,ab. OR common origin.ti,ab. OR bicarotid trunk.ti,ab. OR brachiocephalic trunk.ti,ab. #3 cadaver*.ti,ab. OR autopsy.ti,ab. OR dissection.ti,ab. OR embalmed.ti,ab. #4 morphometr*.ti,ab. OR measurement*.ti,ab. OR diameter*.ti,ab. OR calliper*.ti,ab. OR caliper*.ti,ab. 1 AND 2 AND 3 AND 4	0
			Total records identified	191
